# Development of methodologies for virus detection in soybean and wheat seeds

**DOI:** 10.1016/j.mex.2016.01.005

**Published:** 2016-01-16

**Authors:** Stephanie R.A. Botelho, Thais P. Martins, Macária F. Duarte, Andreza V. Barbosa, Douglas Lau, Fernanda R. Fernandes, Marcio M. Sanches

**Affiliations:** a Embrapa Genetic Resources and Biotechnology, Brasilia, DF, Brazil; b Embrapa Trigo, Passo Fundo, RS, Brazil; c Embrapa Plant Quarantine, Brasilia, Brazil

**Keywords:** SMV, WSMV, Quarantine, Seed certification, RT-PCR, qPCR

## Abstract

Seeds that contain large amounts of oil, starch, fibers and phenols are the most difficult tissues for RNA extraction. Currently, there are some reports of virus detection in seeds using commercial kits for RNA extraction. However, individual seeds were used, which may not be always suitable for analyses that deal with large amounts of seeds. Sangha [1] described a simple, quick and efficient protocol for RNA extraction and downstream applications in a group of seeds of jatropha (*Jatropha curcas*), mustard (*Brassica* sp.) and rice (*Oryza sativa*). We tested this protocol for soybean (*Glycine max*), maize (*Zea mays*), wheat (*Triticum aestivum*) and triticale (×*Triticosecale*) seeds and further reverse transcription PCR (RT-PCR)/quantitative real-time PCR (qPCR) in order to have a faster and more practical method for virus detection from seeds than the traditional scheme of seed planting and subsequent Elisa/RT-PCR from leaves. The essential points in the method are:

• Some modifications in the protocol [1] were done in order to increase performance: Wheat and triticale seeds are incubated with water prior to maceration. An amount of 1.2 g of dry soybean seeds is used to maceration.
• RT-PCR is used for detection of *Wheat streak mosaic virus* from wheat seeds and RT-qPCR for detection of *Soybean mosaic virus* from soybean seeds.
• The method may be tested for other viruses, however, pre-validation will be needed.

## Graphical abstract

**Figure:**
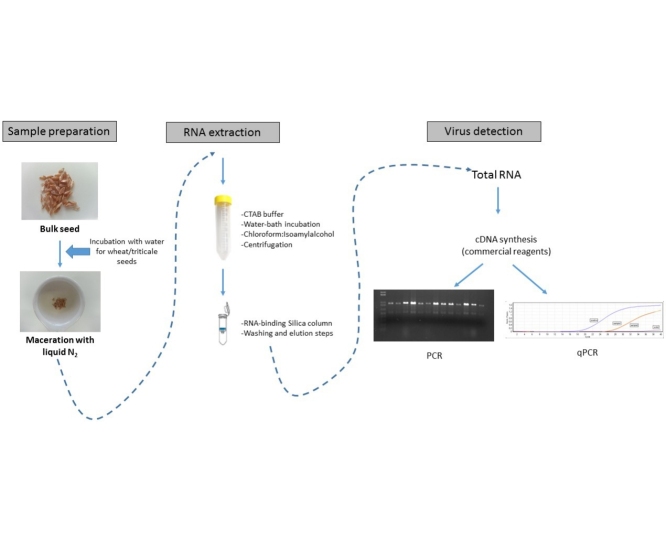

## Methods detail

The protocol described for RNA extraction of immature seeds of jatropha (*Jatropha curcas*), mustard (*Brassica* sp.) and rice (*Oryza sativa*) seeds is a simple, quick and efficient protocol for RNA extraction and downstream applications [1]. To use it for virus detection in soybean (*Glycine max*) and wheat (*Triticum aestivum*) seeds, some improvements are needed to be done in the maceration step. Two viruses of quarantine importance, *Soybean mosaic virus* (SMV), family *Potyviridae*, genus *Potyvirus* [2] and *Wheat streak mosaic virus* (WSMV), family *Potyviridae*, genus *Tritimovirus* [3], [4] were chosen to check if the protocol is suitable for application in their identification in seeds. The molecular tools: RT-PCR test for WSMV detection [3] and the RT-PCR for SMV detection [5] adapted to RT-qPCR were compared to other techniques and selected to perform virus detection with the RNA obtained from seeds.

### Sample preparation

Soybean plants were mechanically inoculated with an isolate of SMV (named 165.09 GenBank: KC331990) intercepted at Plant Quarantine Laboratory of Embrapa Genetic Resources and Biotechnology. Wheat plants of cv. Guabiju were also mechanically inoculated with WSMV (isolate 915 GenBank: KC152463.1 from Passo Fundo, Brazil). Plants were cultivated in greenhouses to obtain seeds. Seeds were harvested and stored at 4 °C until the maceration procedure began. In addition, seeds of soybean (several accessions), wheat cv. BRS Guabiju and BRS Guamirim, triticale cv. BRS Saturno and BRS Ulisses and maize hybrid HS201 from healthy plants were collected for RNA extraction procedure. Prior to maceration, the wheat and triticale seeds were incubated with water in order to soften the tissues.

### Maceration

(1) Put an amount of 0.5 g of wheat/triticale seeds into a mortar and subsequently cover the seeds with about 20 mL of distilled water. Cover the mortar with a paper towel and wait at least 12 h at room temperature to start the maceration in order to allow the seeds to absorb water.
(2) Pulverize 1.2 g of dry soybean seeds, 0.5 g of dry maize seeds and the previously incubated wheat and triticale seeds in liquid nitrogen. Put the seed powder into 50 mL pre-chilled polypropylene (Falcon) tube.

### RNA extraction

(3) In a fume hood, add 5 mL of pre-heated (65 °C) total RNA extraction buffer {2% (w/v) CTAB, 2% (w/v) polyvinylpyrrolidone (PVP-40), 100 mM Tris HCl (pH 8.0), 25 mM EDTA, 2 M NaCl, 0.1% spermidine and 2% β-mercaptoethanol} to the frozen samples and keep them in a water bath (65 °C) for 30 min, vortexing every 5 min.
(4) After incubation, add an equal volume of Chloroform:Isoamyl alcohol (24:1) to each sample, vortex samples for 30 s and centrifuge them at 10,000 × *g* for 20 min at 4 °C.
(5) Transfer the aqueous supernatant (1 mL/tube) above the white phase to 2.0 mL RNase-free microcentrifuge tubes and add an equal volume of Chloroform: Isoamylalcohol. Mix with vortex and centrifuge in a desktop centrifuge at 10,000 × *g* for 10 min at 4 °C.
(6) Transfer the supernatant (1.0 mL), without touching the white layer, to RNase-free 1.5 mL microcentrifuge tubes. Add 0.5 mL of 96–100% ethanol.

### Purification

(7) Immediately, load the supernatant-ethanol mixture in the RNA binding columns of RNeasy Kit Qiagen^®^ (0.75 mL/column), skipping the filtration step. Spin at 10,000 × *g* for 30 s at room temperature.
(8) Load leftover samples in the same columns to process the entire sample.
(9) Follow the subsequent steps of kit protocol to wash and desalt the samples bound with the silica membrane of the column.
(10) Elute the RNA from each column using 50 μL of RNase-free water and store at −80 °C.

### RNA yield, quality and RT-PCR/qPCR conditions

**Fig. 1:**
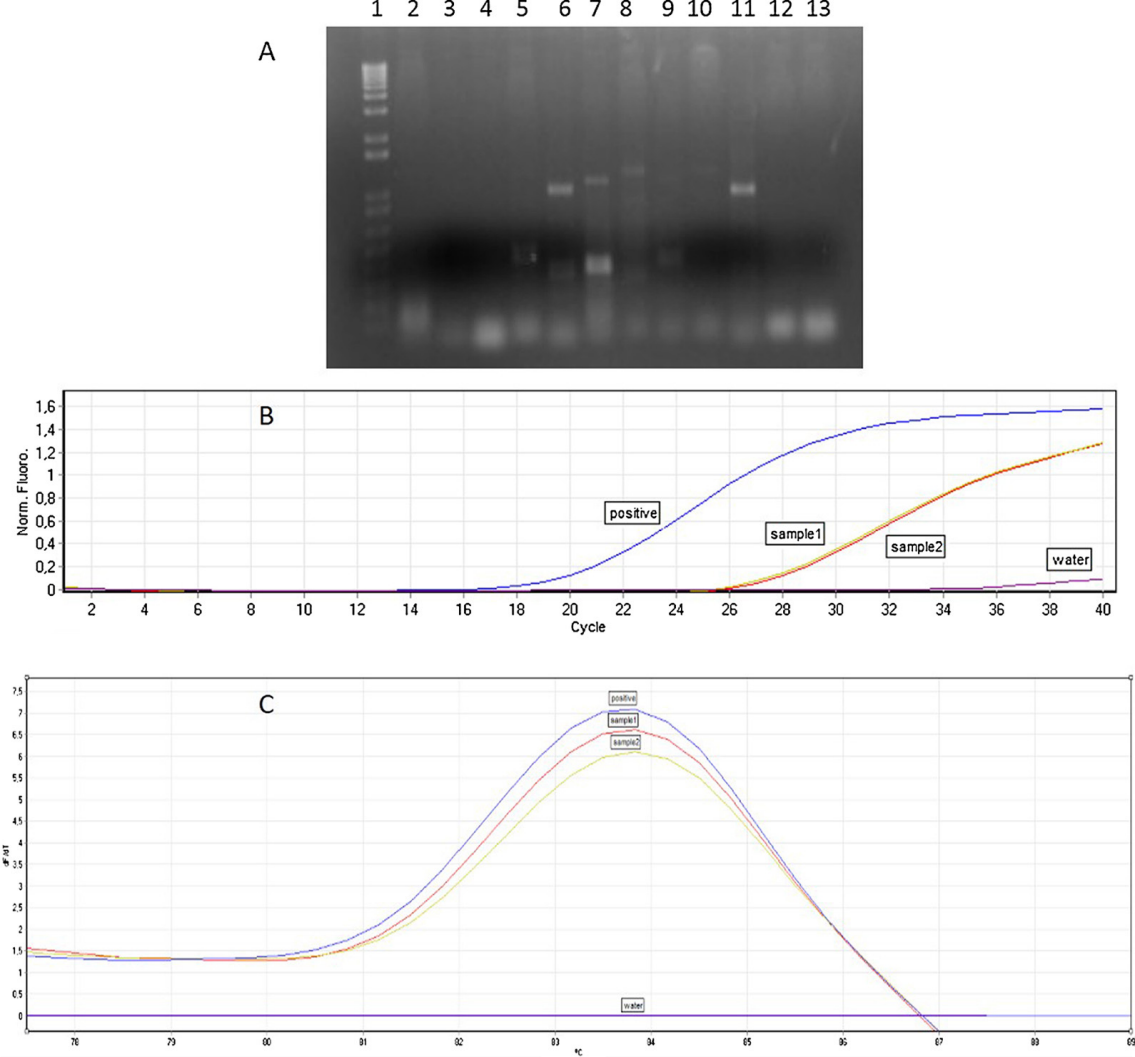
(A) Agarose gel showing amplification for WSMV from RNA extracted from wheat seeds. 1: 1 Kb Plus DNA ladder (Invitrogen); 2–5 and 7–10: WSMV-Negative samples; 6: WSMV-positive sample; 11: Positive-cloned CP fragment of WSMV; 12: wheat healthy seed; 13: negative water control. (B) Amplification profile of qPCR for SMV from RNA extracted from soybean seeds. Sample 1 and 2: SMV-positive samples. Positive: positive control. Water: negative water control. (C) Melting curve analysis for SMV-positive samples.

Verify RNA concentration and quality with a Nanodrop 2000 microvolume spectrophotometer (Thermo Scientific). Perform reverse transcription reactions with MMLV Reverse Transcriptase (Invitrogen) using oligodT or specific primers following the manufacturer's instructions. With the cDNA obtained, perform a PCR for WSMV as follows: 1× PCR buffer (Qiagen), 0.2 mM dNTP, 0.2 μM WSMVf primer (5′-TCGAGTAGTGGAAGCACTCA-3′), 0.2 μM WSMVr primer (5′-CCTCACATCAT CTGCATCAT-3′) [3], 2.25 mM MgCl_2_, 1 U *Taq* DNA polymerase, 1 μL cDNA and RNAse-free water to complete 50 μL. Carry out PCR with an initial denaturation step at 94 °C, 5 min; then, 40 cycles of denaturation (94 °C, 30 s), annealing (57 °C, 1 min) and elongation (72 °C, 1 and a half minutes) and a final step of elongation for 10 min. For SMV perform a qPCR as follows: 1× Rotor Gene SYBR Green Master Mix (Qiagen), 1 μM of each primer SMVcpf (5′-CAAGCAGCAAAGATGTAAATG-3′)/SMVcpr (5′-GTCCATATCTAGGCATATACG-3′) [5], 5 μL of cDNA (previously 10× diluted) and RNAse-free water to complete 25 μL. Carry out the qPCR with an initial denaturation step at 95 °C, 5 min; then, 45 cycles of denaturation (95 °C, 5 s) and annealing-elongation (60 °C, 10 s). In addition, detection of viruses is confirmed by agarose gel electrophoresis for WSMV, amplification profile and melting curve analysis of qPCR for SMV. The PCR products of the expected size (469 bp for SMV and 948 bp for WSMV) can be sequenced to confirm the identity of the virus. The quality of RNA obtained from healthy and infected seed samples, their concentrations and results of virus detection are summarized in Table 1 and Fig. 1.

### Validation

**Fig. 2:**
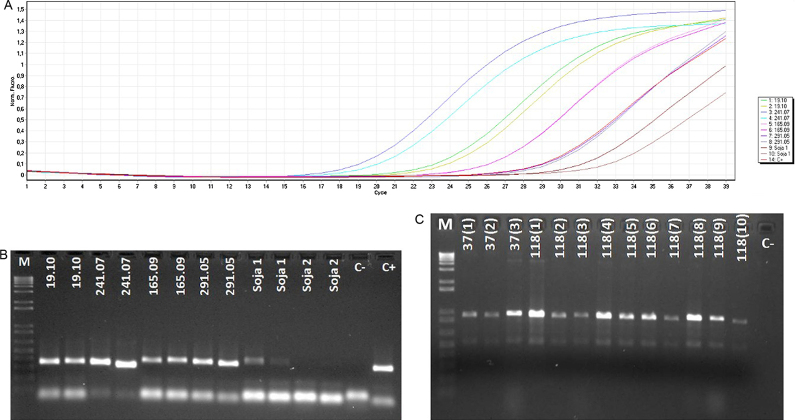
(A) Amplification profile of validation experiment for SMV qPCR test. (B) Agarose gel of qPCR products for SMV validation experiment. (C) Agarose gel of validation experiment for WSMV PCR test. The codes in gel represent samples, except M: 1 Kb Plus DNA ladder (Invitrogen); C−: negative water control; C+: positive control.

In order to use the method for routine procedures, we validated the method according to EPPO [6] guidelines. Because only one sample of wheat seeds was positive to WSMV, thirteen recombinant plasmids with the coat protein fragment of WSMV were diluted up to 10^−3^ in wheat/triticale seed RNA to simulate the conditions of the test (presence of potential inhibitors for PCR test in the seeds). Two independent experiments with conventional PCR were performed as described above. Five isolates of SMV had the RNA extracted from leaves using the RNeasy mini kit (Qiagen) and the cDNA prepared as described above. The cDNAs were mixed with soybean seed RNA (due to the presence of potential inhibitors for PCR test in the seeds) and diluted up to 10^−2^ for subsequent qPCR. Two independent experiments were performed for duplicate samples with the same conditions previously described. Results are available in Table 2 and Fig. 2. In addition, the seed samples with positive results for WSMV and SMV were submitted to the traditional quarantine scheme of planting and Elisa test from leaves and similar results were obtained (Table 2).

## Additional information

### Background information

About 230 plant virus and viroid that cause diseases on plants are seed-transmitted [7]. There are economically important virus species, such as SMV and WSMV, which are transmitted through soybean and wheat seeds respectively. Traditionally, the scheme for detection of SMV and WSMV includes seed planting, in order to observe typical symptoms and the Elisa tests from leaf samples or reverse transcription PCR (RT-PCR) with specific primers [4], [5], [8]. This scheme is time-consuming (3–4 weeks to observe symptoms) and might not have enough sensibility to detect the viruses in low concentrations. Currently, there are some reports of detection of RNA viruses through commercial kits in tomato and pepper seeds [9], [10]. However, individual seeds were used, which may not be always suitable for quarantine or seed certification analyses that deal with large amount of seeds. In addition, soybean seeds are rich in oil and wheat seeds are rich in starch. Our previous analysis using different commercial kits for these seeds showed low quantity and quality of RNA.

The results showed that the concentration (in ng) of all samples was within the acceptable range with good quality (low concentration of proteins), varying among samples. This variation could had occurred due to the nature of samples (storage conditions, age of seeds, among others) and also occurs when commercial kits are used for RNA extraction from more suitable tissues, such as leaves. The virus detection was performed and results were the same as obtained by planting-Elisa method. No false-positive result was obtained and the false-negative results were in the acceptable range [6]. Unfortunately, non-specific bands, different in size compared to positive control, were visualized in the PCR for WSMV (Fig. 1A, lanes 7 and 8). These bands were sequenced and had the highest identity with plant genes. However, it was not possible to improve the specificity of the method without an increase in the level of false-negative results. On the other hand, the bands that were slightly different in size in the qPCR for SMV (Fig. 2A) may reflect the genetic differences in the virus population. The presence of recombination events and mutations in the genome of the SMV are considered responsible for the emergence of virulent strains [11]. In addition, defective virus frequently occurs in certain proportion in a viral population [12].

The total time for analysis, including cDNA synthesis and qPCR, might be less than seven hours (nine hours for conventional PCR). This case study reveals the potential to be an alternative to the current scheme of planting and testing from symptomatic tissues. A single sample from a batch of seed could be tested against certain viruses, reducing the time and the space required for analysis. This is especially important for analysis in places where it is impossible to plant the seeds, or when the time for detection is short, like for inspection in harbors, airports or border areas. Sometimes seeds present reduced viability, especially when infected by viruses or other pathogens, and the planting is useless for analysis.

The seeds infected by SMV and WSMV collected for RNA extraction did not show symptoms such as mottling or reduced size. Due to the potential presence of other virus species transmitted by seeds in soybean, like *Cowpea mild mottle virus* (genus *Carlavirus*), *Tobacco ringspot virus* (genus *Nepovirus*), *Tobacco streak virus* (genus *Ilarvirus*) and in wheat, like *Barley stripe mosaic virus* (genus *Hordeivirus*) among others [7], the extraction of RNA from seeds may be very useful for seed certification and quarantine. To improve this approach, other specific tests against a broad range of seed-transmitted viruses have to be developed and/or validated in order to keep the same reliability. In these experiments, the amount of 1.2 g corresponded to approximately 8–10 soybean seeds. The amount of 0.5 g corresponded to approximately 20–25 entire wheat seeds or 40–50 longitudinally cut half seeds.

## Figures and Tables

**Table 1:** Concentration and quality of RNA extracted from soybean, wheat, triticale and maize seeds.

Sample	Concentration	*A*_260_/*A*_280_	Remarks^a^
Maize HS201	67.3 ng/μl	2.05	Healthy seeds
Triticale cv. BRS Ulisses	164.8 ng/μl	2.08	Healthy seeds
Wheat cv. BRS Guamirim	53.9 ng/μl	2.01	Healthy seeds
Triticale cv. BRS Saturno	13.4 ng/μl	2.23	Healthy seeds
Soybean1	378.7 ng/μl	2.11	Healthy seeds
Soybean2	803.5 ng/μl	2.08	SMV-positive seeds
Soybean3	505.4 ng/μl	2.11	SMV-positive seeds
Soybean4	27.5 ng/μl	2.02	SMV-negative seeds
Soybean5	102.4 ng/μl	2.00	SMV-negative seeds
Wheat cv. BRS Guabiju1	157.3 ng/μl	2.09	Healthy seeds
Wheat cv. BRS Guabiju2	15.7 ng/μl	2.14	WSMV-negative seeds
Wheat cv. BRS Guabiju3	248.4 ng/μl	2.10	WSMV-negative seeds
Wheat cv. BRS Guabiju4	11.3 ng/μl	2.10	WSMV-positive seeds
Wheat cv. BRS Guabiju5	87.3 ng/μl	2.06	WSMV-negative seeds
Wheat cv. BRS Guabiju6	63.8 ng/μl	1.94	WSMV-negative seeds
Wheat cv. BRS Guabiju7^b^	340.2 ng/μl	2.06	WSMV-negative seeds

a Healthy seeds: collected from healthy plants; SMV and WSMV positive or negative seeds: collected from plants inoculated with viruses.

b Cut transversally half seeds with the embryo.

**Table 2:** Validation experiments for WSMV and SMV diagnosis.

Sample	Experiment 1^a^	Experiment 2^b^
WSMV (conventional PCR)	13/17	13/13
SMV (real-time PCR)	10/10	10/10
Negative water control (both assays)	0/3	0/3
Healthy seed (both assays)	0/6	0/6
Elisa test SMV (from leaves)	5/5	–
Elisa test WSMV (from leaves)	1/1	–

a Total of positive samples/total of samples tested.

b Only performed for PCR based tests.
